# Giant Sex Chromosomes in *Omophoita* Species (Coleoptera, Chrysomelidae): Structural and Evolutionary Relationships Revealed by Zoo-FISH and Comparative Genomic Hybridization (CGH)

**DOI:** 10.3390/insects14050440

**Published:** 2023-05-04

**Authors:** Jhon A. D. Vidal, Francisco de M. C. Sassi, Renata L. R. de Moraes, Roberto F. Artoni, Thomas Liehr, Marcelo B. Cioffi, Mara C. de Almeida

**Affiliations:** 1 Laboratório de Citogenética de Peixes, Departamento de Genética e Evolução, Universidade Federal de São Carlos (UFSCar), Rodovia Washington Luiz Km. 235, C.P. 676, São Carlos 13565-905, Brazil; jhonalex279@gmail.com (J.A.D.V.); francisco.sassi@hotmail.com (F.d.M.C.S.); rlrdm.rm@gmail.com (R.L.R.d.M.); mbcioffi@ufscar.br (M.B.C.); 2 Laboratório de Genética e Evolução, Departamento de Biologia Estrutural Molecular e Genética, Universidade Estadual de Ponta Grossa (UEPG), Av. Carlos Cavalcanti, 4748, Ponta Grossa 84030-900, Brazil; rfartoni@gmail.com (R.F.A.); almeidamara@uol.com.br (M.C.d.A.); 3 Institute of Human Genetics, University Hospital Jena, 07747 Jena, Germany

**Keywords:** beetles, achiasmatic, Oedionychina, WCP

## Abstract

**Simple Summary:**

Unlike most beetles that have small XY sex chromosomes, the Oedionychina group (Coleoptera, Alticinae) has atypical sex chromosomes, which are called giant sex chromosomes because they are much larger than the autosomes. There is little knowledge about the origin and differentiation of these sex chromosomes. We used *Omophoita* as a model to investigate the evolution of such giant sex chromosomes, using comparative genomic hybridization (a technique that compares genomes of species by co-hybridizing into chromosomes) and chromosome painting with a probe derived from both X and Y sex chromosomes. It was verified that there is a high degree of similarity between the genomes of male and female *O. octoguttata*, with a sex-specific region on the Y chromosome. The comparison between the genomes of the species showed that their autosomes are quite different from each other. However, when comparing the sex chromosomes by chromosome painting, it was shown that the sex chromosomes are similar. Based on these results, it can be inferred that the *Omophoita* sex chromosomes arose and evolved by the same process, and that due to the high degree of similarity between them, there has not yet been enough time for great divergence between the sex chromosomes.

**Abstract:**

The beetles of the subtribe Oedionychina (Chrysomelidae, Alticinae) are the only ones that have the atypical giant and achiasmatic sex chromosomes, which are substantially larger than the autosomes. Previous cytogenetic analyses suggest a large accumulation of repetitive DNA in the sex chromosomes. In this study, we examined the similarity of X and Y chromosomes in four *Omophoita* species and compared genomic differentiation to better understand the evolutionary process and the giant sex chromosomes origin. Intraspecific genomic comparation using male and female genomes of *O. octoguttata* and interespecific analyses using genomic DNA of *O. octoguttata*, *O. sexnotata*, *O. magniguttis*, and *O. personata* were performed. In addition, whole chromosome painting (WCP) experiments were performed with X and Y chromosome probes of *O. octogutatta*. CGH analysis revealed great genomic similarity between the sexes and a sex-specific region on the Y chromosome, and interspecific analysis revealed a genomic divergence between species. In contrast, WCP results revealed that the sex chromosomes of *O. octoguttata* have high intra- and interspecific similarity with the studied species. Our data support a common origin under the canonical evolution of the sex chromosomes in this group, as they have high genomic similarity between them.

## 1. Introduction

With 380,000 described species, the order Coleoptera is the most diverse group in the class Insecta [1]. Beetles are notable for their enormous diversity among groups, as well as their ability to live in different habitats and feed on a variety of organic materials [2]. With approximately 4900 species, they represent one of the insect groups with the largest number of species analyzed cytogenetically [3]. The diploid number and chromosome structure of such species vary greatly, ranging from 2n = 4 (*Chalcolepidius zonatus*) [4] to 2n = 72 (*Xanthogaleruca luteola*) [5]. Despite such a karyotypic variability, some cytogenetic analyses consider 2n = 20 = 9II + Xyp as the basal karyotype for Polyphaga, with nine pairs of autosomes and an XY sex system, in which the X chromosome is considerably larger than the Y [6,7]. The Xyp system is named so due to the fact that the sex chromosomes do not recombine during meiosis and stay separated from each other in a shape similar to that of a parachute [3].

Aside from the typical Xyp sex-determination system found in Coleoptera, some derivations of this system can also be observed, such as the XY, Xy, NeoXY, X0, and XnYn systems [3,8,9,10]. These variants differ from Xyp in both origin and size, in addition to the fact that they recombine at meiosis I. The XY system has similar sizes between X and Y; the Xy has an X that is significantly larger than the Y; the NeoXY has a possible formation derived from the fusion of a sex chromosome with an autosome; the X0 differs by the loss of the Y, and finally, there is the XnYn, being characterized by the formation of multiple sex chromosomes [3,10].

Among the groups of Coleoptera that have been cytogenetically analyzed, the flea beetles Alticinae are considered derived, since they have a karyotype that varies in terms of chromosome number and sex determination system [11,12]. Within Alticinae, “Oedionychina” (Oedionychini *sensu* Chapuis 1875) is the subtribe whose cytogenetics have been most studied, given the presence of giant sex chromosomes [13,14,15,16,17,18,19,20,21,22,23,24,25,26,27]. However, the group’s phylogenetic relationships have not been resolved because of their diversity; most studies are limited and do not include all available species [28,29]. Molecular investigations based on multi-locus sequencing data suggest that the previously recognized Alticini is a non-monophyletic group, and reorganize the genera between monophyletic Alticinae and Galerucinae [29,30]. Thus, the only data involving this subtribe Oedionychina come from Bechyné and Springlova de Bechyné [31], who divided Oedionychini into two subtribes, Oedionychina and Disonychina, based on morphological data and the number of spermatozoa per bundle. Mitochondrial and nuclear DNA-based phylogeny suggests a suprageneric classification as Oedionychites, including the genera *Omophoita*, *Physodactyla*, *Oedionychis*, *Eutornus*, *Physonychis*, *Lithonoma*, and *Physoma* [32]. For the present investigation, the morphological subtribe classification of Oedionyochina is adapted since most cytogenetics studies, especially on Neotropical flea beetles, still accept this as the most reliable phylogenetic classification.

Oedionychina is an insect group known to have giant sex chromosomes that are significantly larger than autosomes, making it an exceptional group [33]. Most Oedionychina species present a conserved diploid number (2n = 22 = 10II + X + y) [7,11,13,14,15,27,34]. Conventional cytogenetic analysis (Giemsa stain, Ag-NOR) and the mapping of the ribosomal cluster rDNA 45S and 5S have shown that there is little variation between species concerning the loci of these markers [10,13,15,19,34,35]. However, a substantial variation is found among species considering the chromosomal distribution of C-positive heterochromatin and some transposable elements (TEs) [17,18]. However, up to now, fewer studies addressed the origin and differentiation of such atypical sex chromosomes. The insertion and amplification of repetitive DNA, as well as previous research using standard cytogenetics have suggested that sex chromosomes may have experienced a variety of chromosomal rearrangements, which can reduce the homology between the sex chromosomes [13,17,18]. In recent years, there has been a clear trend toward using modern molecular cytogenetic methods in insect research. In particular, chromosomal painting techniques (WCP) and comparative genomic hybridization (CGH) have become more widely used in the area of comparative cytogenetics, providing access to more fine-scale insights into a variety of evolutionary processes [36,37].

Therefore, besides having the conserved karyotype in most species, Oedionychina can be considered an exceptional group for studies on sex chromosome evolution as it is the only group of insects presenting with giant sex chromosomes so far, where the X and Y are larger than autosomes [7]. Research in this group opens new paths for understanding how sex chromosomes differentiate (in size, across species, and in repetitive DNA content), and the evolution of beetles as a whole. However, the information on the number of chromosomes, the existence of the sex chromosome system, and their function during meiosis has been only obtained and explored using traditional methods [13,14,15,16,17,18]. Therefore, this work focuses on the genomic composition of the X and Y chromosomes of four *Omophoita* species named *Omophoita octoguttata* Fabricius, 1775, *Omophoita personata* Illiger, 1807, *Omophoita sexnotata* Harold, 1876, and *Omophoita magniguttis* Bechyné, 1955 by applying a suite of molecular cytogenetic techniques, including the comparative genomic hybridization (CGH) and whole chromosome painting (WCP). The findings helped expand our knowledge regarding the differentiation of this atypical sex chromosome system.

## 2. Materials and Methods

### 2.1. Animals and Chromosomal Preparations

*Omophoita octoguttata*, *O. sexnotata*, *O. magniguttis*, and *O. personata* specimens were collected in Itaiacoca-PR- Brazil (25°07′05.0″ S 49°56′25.3″ W) and stored in the Genetics and Evolutionary Laboratory at the Universidade Estadual de Ponta Grossa. Insect dissection was used for obtaining the mitotic and meiotic chromosomes, following Rosolen et al. [18]. The slides were rinsed with distilled water, stained for 12 min with 3% Giemsa in phosphate buffer pH 6.8, and then air-dried. The Chico Mendes Institute for Biodiversity Conservation (ICMBIO), the System of Authorization and Information about Biodiversity (SISBIO-Licenses No. 15402), and the National System of Genetic Resource Management and Associated Traditional Knowledge (SISGEN ABE8B7D), all in Brazil, granted authorization for the collection of specimens.

### 2.2. Comparative Genomic Hybridization (CGH)

Two different experimental designs were used in this study: (1) In a set of intraspecific comparative experiments, the gDNA of males and females of *O. octoguttata* were co-hybridized against the background of male meiotic chromosomes of *O. octogutatta*. Male- and female-derived gDNA from *O. octoguttata* were labelled using ATTO 550-dUTP, and ATTO 488-dUTP, respectively, both through nick translation (Jena Bioscience, Jena, Germany). We used C0t-1 DNA produced from female gDNA in accordance with Zwick et al. [38] to block common genomic repetitive regions. The final hybridization mixture for each slide (20 µL) was composed of 500 ng of *O. octoguttata* male gDNA, 500 ng of the *O. octoguttata* female gDNA, and 30 µg of unlabeled female-derived C0t-1 DNA from females of *O. octoguttata*, all resuspended together in the hybridization buffer containing 50% formamide, 2 × SSC, 10% SDS, 10% dextran sulfate, and Denhardt’s reagent (pH 7.0); (2) In a set of interspecific comparisons, gDNA of males from *O. octoguttata* was co-hybridized with the gDNA of males of *O. octoguttata*, *O. personata*, *O. magniguttis*, and *O. sexnotata* hybridized against the background of male meiotic chromosomes of *O. octogutatta*. The gDNA of *O. octoguttata* was labeled with Atto 550-dUTP, while the gDNAs of the other species were labeled with Atto 488-dUTP, both through nick translation (Jena Bioscience, Jena, Germany). In all experiments, the final probe cocktail for each experiment was composed of 500 ng of each probe and 15 µg of C0t-1 DNA of the respective individuals, diluted in the same hybridization buffer as described above. FISH experiments were performed using high-stringency conditions in accordance with Sassi et al. [39]. In summary, prior to the hybridization, the slides were aged for two hours at 37 °C, followed by RNAse A (90 min, 37 °C) and pepsin (50 g/mL in 10 mM HCl, 3 min, 37 °C) treatments. Following denaturation in 75% formamide (pH 7.0) in 2 SSC for 3 min at 74 °C, the slides were immediately cooled and dehydrated via a 70% (cold), 85%, and 100% (RT) ethanol series. The hybridization mixture was denatured at 86 °C for 6 min, then cooled at 4 °C for 10 min before being applied to the slides. The hybridization lasted 72 h at 37 °C. After hybridization, post-hybridization washes were performed once in 50% formamide in 2 × SSC (pH 7.0) (44 °C, 10 min) and three times in 1 × SSC (44 °C, 7 min each). Counterstained chromosomes were mounted with antifade containing 1.5 g/mL DAPI (Vector, Burlingame, CA, USA).

### 2.3. Whole Chromosome Painting (WCP)

We isolated fifteen copies of the giant X and Y chromosomes of *O. octoguttata* by glass needle-base microdissection and amplified the DNA using the procedure described in Yang et al. [40]. The obtained probes (named OCT-X and OCT-Y) were then labeled with Spectrum Green-dUTP and Spectrum Orange-dUTP (Vysis, Downers Grove, IL, USA), respectively, in a secondary DOP-PCR, using 1 μL of the primarily amplified product as a template DNA [40]. FISH experiments were performed according to Yano et al. [41], and to block the hybridization of high copy repeat sequences, 20 μg of C0t-1 DNA directly isolated from *O. octoguttata* female genome were prepared in accordance with Zwick et al. [38].

### 2.4. Image Analysis and Processing

For each FISH experiment, at least 30 metaphase spreads were examined in order to validate the 2n, karyotype, and FISH outcomes. Images were captured with a CoolSNAP microscope, an Olympus BX50 (Olympus Corporation, Ishikawa, Japan), and then processed with Image Pro Plus 4.1 (Media Cybernetics, Silver Spring, MD, USA). Adobe Photoshop, version CC 2020 was used to optimize and organize the final photos.

## 3. Results

### 3.1. Detection of the Male-Specific Region by CGH

**Figure 1:**
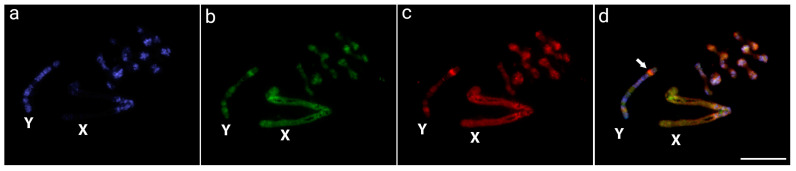
Meiotic chromosome spreads of *Omophoita octoguttata* males after CGH—intraspecific comparisons. Male-derived genomic probe from *O. octoguttata* (**b**), female-derived genomic probe from *O. octoguttata* (**c**). The first column (**a**): DAPI images (blue); Second column (**b**): hybridization pattern using the female-derived probe (green) of *O. octoguttata*; Third column (**c**): hybridization pattern using the male-derived probe (red). The fourth column (**d**): merged images of both genomic probes and DAPI staining, with arrow indicating the male-specific genomic region on Y chromosome. The common genomic regions of both compared karyomorphs are depicted in yellow and XY sex chromosomes are indicated. Bar = 10 μm.

According to the intraspecific CGH experiments performed in *O. octoguttata*, both genome-derived probes preferentially localize in the centromeric and pericentromeric regions of most chromosomes and autosomes, as well as in a great part of the X chromosome. These regions matched the C-banding pattern, indicating their repetitive content. In addition, the Y chromosome stood out with an interstitial signal on its short arms when using the male-specific gDNA probe, suggesting a male-specific area (Figure 1).

### 3.2. Interspecific Comparative Genomic Hybridization (CGH)

**Figure 2:**
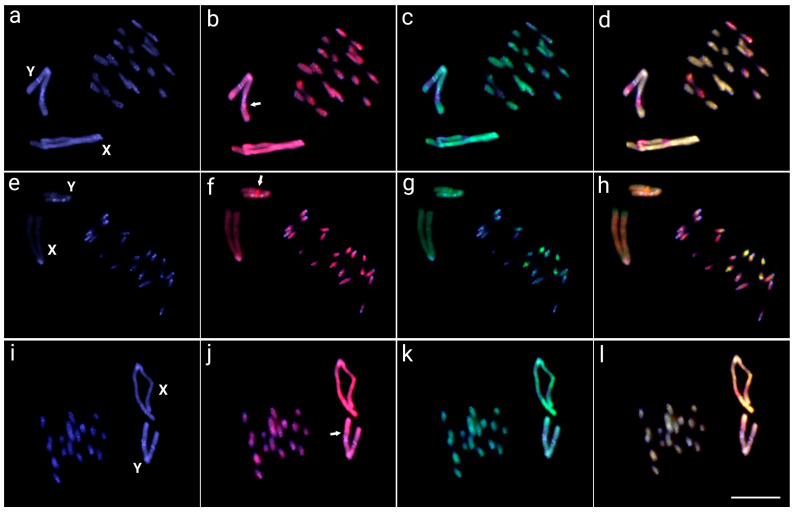
Meiotic chromosome spreads of *Omophoita octoguttata* males after CGH—interspecific comparisons. Male-derived genomic probe from *O. octoguttata* and *O. magniguttis* (**a**–**d**), *O. personata* (**e**–**h**), *O. sexnotata* (**i**–**l**) mapped against male chromosomes of *Omophoita octoguttata*. First column (**a**,**e**,**i**): DAPI images (blue); Second column (**b**,**f**,**j**): hybridization pattern using the male-derived probe (red) of *O. octoguttata*; Third column (**c**,**g**,**k**): hybridization pattern using the male-derived probe of each other compared species (green). Fourth column (**d**,**h**,**l**): merged images of both genomic probes and DAPI staining. The common genomic regions of both compared karyomorphs are depicted in yellow, XY sex chromosomes are indicated, and arrows point to the sex-specific region on Y chromosome. Bar = 10 μm.

The interspecific CGH performed in the meiotic chromosomes of *O. octoguttata* compared with the genomic DNA of *O. octoguttata*, *O. personata*, *O. sexnotata*, and *O. magniguttis* demonstrated a high genomic similarity in the repetitive content among species (Figure 2). The sex chromosomes of *O. octoguttata* with *O. magniguttis* demonstrated a high similarity, with only some species-specific signals in both X and Y chromosomes. The X chromosome has two *octoguttata*-specific signals in the long arm, one in the interstitial region and the other in the terminal region, whereas the Y chromosome has two specific signals, one in the terminal region of the short arm and the other in the interstitial long arm (Figure 2a–d).

The hybridizations using *O. personata* gDNA revealed differences in autosomes with the *O. octoguttata* genome, with some autosome pairs highly shared among species and autosome pairs weakly shared. Most autosomes have more genomic similarity with the *O. octoguttata* genome, with about three pairs sharing repetitive contents with the *O. personata* genome. Additionally, the Y chromosome presented a stronger hybridization pattern to the long arms with the *O. octoguttata* probe (Figure 2e–h). On the other hand, the comparison with the genomic DNA of *O. sexnotata* shows a high degree of similarity among species, with co-hybridization of both genomic probes in all autosomes and sex chromosomes. However, a small *octoguttata*-specific region X sex chromosome was observed (Figure 2i–l).

### 3.3. Whole Chromosome Painting (WCP)

**Figure 3:**
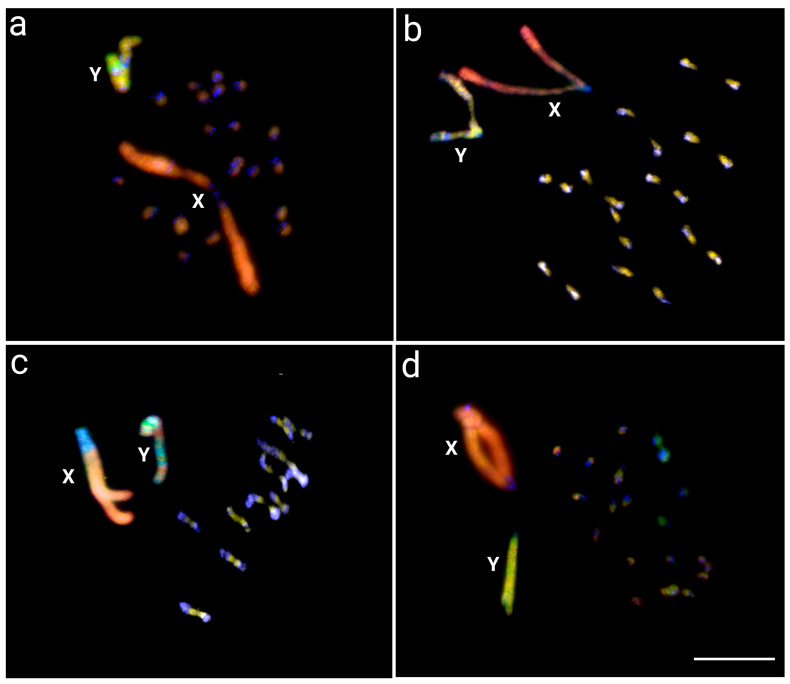
Meiotic chromosome spreads of *O. octoguttata* (**a**), *O. sexnotata* (**b**), *O. personata* (**c**), and *O. magniguttis* (**d**). After intra- and interspecific WCP comparisons, using the X-derived chromosome probe from *O. octoguttata* (green) and Y-derived chromosome probe from *O. octoguttata* (red).

The OCT-X and OCT-Y probes showed signals on both sex chromosomes, with small scattered signals on the autosomes of all species analyzed (Figure 3). The hybridization pattern observed in the Y chromosome of *O. octoguttata* (Figure 3a), *O. sexnotata* (Figure 3b), and *O. personata* (Figure 3c) consists of little overlapped regions of both WCP probes along the entire chromosome and a small OCT-Y-specific region near the centromere, in addition to several OCT-X probed blocks. On other hand, *O. magniguttis* (Figure 3d) presents its Y chromosome almost exclusively marked with the OCT-Y.

## 4. Discussion

*Omophoita* and other Oedionychina species have the largest disparity in their sex chromosome sizes, where both X and Y are more than 10 times larger than the autosomes [13,23]. Although “giant” sex chromosomes have been described in other plant and animal groups (e.g., [42,43,44,45]), neither has that incredible size disparity. Here, our study demonstrated that the giant XY sex chromosome system present in four *Omophoita* species had a common origin, considering the homology found in all species using the X and Y chromosome painting probes from *O. octoguttata*. Moreover, distinct similar hybridization patterns were confirmed for the X and Y chromosomes in CGH and WCP studies among all species, indicating that these sex chromosomes had not undergone fast discordant differentiation.

The processes influencing the differentiation and evolution of sex chromosomes are closely related to heterochromatinization and differential accumulation and/or amplification of repetitive DNA sequences [46,47]. This is also true for the giant sex chromosomes present in *Omophoita* species, which usually present pericentromeric and interstitial heterochromatic regions [13]. The diploid numbers found in the species herein analyzed are in accordance with the previous ones described by Almeida, Campaner, and Cella [13]. Most Oedionychina species maintain the same karyotype composition with few exceptions [11,16,26,27]. All Oedionychina species share giant sex chromosomes, and there are some variations of this system, such as for *Asphaera*, which has just one giant Y chromosome and many X chromosomes (Xn + Y) [26].

Our findings revealed that *O. octoguttata* had substantial intra- and interspecific genomic divergence when compared to *O. personata*, *O. sexnotata*, and *O. magniguttis*. Nevertheless, the sex chromosomes of all species showed conservation of their general genomic composition, with minor species-specific regions. Although not including all *Omophoita* species that were investigated here, previous molecular phylogenetic reconstructions of flea beetles suggest that the Oedionychina group has diverged in the middle Cretaceous (~100 mya) [29] even though *Omophoita* is considered the most derived genus [48]; this age can also be deduced as the origin of this giant sex chromosome system since it is shared by all subtribe members [26]. Older achiasmatic sex chromosome systems are observed in other Arthropods, such as Hemiptera true bugs [49,50], while for the fly *Drosophila albomicans*, the switch from chiasmatic to achiasmatic is dated to 0.55 mya [51].

The evolution of sex chromosomes in Coleoptera is a recurring topic in the literature. Most species present a Xy sex chromosome system [52] and share an ancestral X chromosome [53] but with several morphological patterns. Furthermore, the derivations of this basal system may have resulted from fusion, fission, and other chromosome rearrangements within this order, showing a high potential for Y loss and gain [9,10,52,54]. This suggests that especially X–autosome fusions were responsible for the high turnover observed in beetle species.

Sex chromosome–autosome fusion processes may be linked to the origin of the Oedionychina sex chromosomes, as this mechanism is widely described in Coleoptera [9,10,52]. Both X and Y *Omophoita* sex chromosomes are thought to have a recent origin and are from the same autosomal pair, retaining a significant level of molecular similarity (Figure 1 and Figure 3). On the other hand, because changes in genomic architecture are known to accumulate over time, sex chromosomes that have developed over a long period can have a considerably higher amount of genomic divergence [45,55,56,57,58]. The fusion process is supported by the presence of interstitial telomeric site (ITS) signals found in *Alagoasa* [15] and *Omophoita* [27] species, indicating that chromosome fusions may have occurred before the growth of Oedionychina giant sex chromosomes. Indeed, high genomic similarities between the similarly sized X and Y chromosomes of *Omophoita* species, as demonstrated by our WCP research, support this hypothesis (Figure 3). This process of fusion between Oedionychina’s sex chromosomes may be related to the decrease in chromosome number in this group because in Chrysomelidae, having 2n = 24 chromosomes is considered a plesiomorphic feature, whereas in Oedionychina, 2n = 22 is considered a plesiomorphic feature [27,33].

Many vertebrate species, including mammals, birds, reptiles, amphibians, and fish, currently possess large sex chromosomes. However, only the sex-specific chromosome (Y or W) has such an abnormally large size [44], unlike the Oedionychina group where both the X and Y chromosomes are giant and exhibit the most discrepant size of sex chromosomes compared to autosomes. The *Microtus* mole vole species, which also have enlarged sex chromosomes, represent the most comparable cases [59,60,61]. Even though *Microtus* and Oedionychina share large sex chromosomes, their evolutionary pathways are completely distinct. According to analyses of heterochromatin, repetitive DNA mapping, and X and Y chromosome painting, the X and Y chromosomes of the *Microtus* species exhibit high levels of intra- and interspecific divergence [43,59,61,62,63]. On the other hand, the *Omophoita* ones showed a high degree of sex-genetic similarities (Figure 2 and Figure 3). This suggests that in addition to the process of fusion of sex chromosomes with autosomes, *Omophoita* sex chromosomes may have evolved through the standard sex chromosome evolutionary process, in which the autosomal pair acquires a sex-determining factor, an inversion process reduces recombination, followed by the accumulation of repetitive sequences and corresponding amplifications, causing such a chromosome expansion [44,45]. According to Chalopin et al. [47], before the differentiation of sex chromosomes and the low degree of homology between them, there is an accumulation of repetitive sequences and the growth of these chromosomes. Previous data from Mello et al. [17] and Rosolen et al. [18], which showed that the sex chromosomes of *Omophoita* have accumulated distinct repetitive DNA classes during their evolutionary history, support this process of enlarging sex chromosomes from the accumulation of repetitive DNA. Moreover, we use CGH to show the locations on the Y chromosome of *O. octoguttata*, where there are likely sex-specific (Figure 1, arrow). Such a region exhibits differences when compared to the genomes of other species, accumulates male-derived gDNA probes, and is bounded by heterochromatin (Figure 2). Many insects have sex chromosomes, which they use to genetically determine their sexes, but the underlying molecular processes are extremely variable, with different sex-determinant variables depending on the species (e.g., [64,65,66,67,68]).

## 5. Conclusions

Several studies in different animal groups have provided some information on what triggers the sex chromosomes to grow, with a sense of the primary cause being the enlargement of the sex-specific (W or Y) chromosome by the accumulation of repetitive DNA sequences (reviewed in [44]). However, *Omophoita* species are a rare case in which both X and Y chromosomes are considered giant, providing a fresh paradigm for understanding the evolutionary dynamics that play a key role in shaping chromosome architecture. Here, our results support the addition of new information for comprehending the evolution of such an unusual sex chromosomal system present in *Omophoita* and demonstrated the following: (i) That the giant XY sex chromosomes had a single origin and so likely originated in the last common ancestor of *Omophoita* species; (ii) The capacity to identify a male-specific region and the stability of sex chromosome genomic content across different congeneric species. In light of these findings, we emphasize the importance of employing insect species as models for research into the evolutionary factors that drove the formation of the animal sex chromosomes. Future research will use high throughput sequencing in microdissected sex chromosomes to further our understanding of sex determination in these species and its likely link to the speciation process. Indeed, some members of Chrysomelidae have the habit of feeding on pollen and flowers, such as Sagrinae, others on seeds such as Bruchinae, which can cause direct damage and loss to agricultural production. However, the vast majority feed on leaves, with damages reported to improper commercially target plants [69]. The knowledge of chromosomes and sex-determination systems in agricultural pests can be important to biological control, especially in the production of sterile insects [70].

## Data Availability

The data presented in this study are available on request from the corresponding author.
